# Modelling and impact of tensiometer plate geometry and sample volume on biosurfactant surface activity assessment

**DOI:** 10.1016/j.heliyon.2024.e38325

**Published:** 2024-09-23

**Authors:** N. Russo-Martínez, X. Vecino, A.B. Moldes, J.M. Cruz

**Affiliations:** Chemical Engineering Department, School of Industrial Engineering – CINTECX, University of Vigo, Campus As Lagoas-Marcosende, 36310, Vigo, Spain

**Keywords:** Wilhelmy method, Surface tension, Biosurfactant, Plate, Geometry

## Abstract

Biosurfactants are molecules with hydrophilic and hydrophobic moieties with the capacity to reduce the surface tension of water. Given the limited quantity of biosurfactant extracts in laboratories, it is recommended to use equipment that requires minimal sample quantities for detecting the presence of biosurfactants. In this work, commercial glycolipids biosurfactants (rhamnolipids or sophorolipids) were diluted in water and subjected to different analyses to obtain their minimum surface tension (ST) reduction and their critical micellar concentration (CMC). The independent variables of the study were: the geometry of platinum plate (rectangular or cylindrical), the sample volume (2, 4 and 20 mL) and the container material consisting of either glass or polytetrafluoroethylene (PTFE). The variation of ST with biosurfactant concentration was studied based on the isotherm model proposed by Li & Lu. It was observed that the profile of ST values did not vary so much using the different independent variables described, observing that platinum rectangular plate can be used for volumes of 4 mL biosurfactants instead of cylindrical plate usually recommended for volumes lower than 20 mL, the container material was also not significant based on the Pearson and Spearman statistical treatment. Moreover, well-fitting regression model results were obtained for a non-commercial biosurfactant extract obtained from a residual stream of the dairy industry, predicting values close to the observed data.

## Graphical abstract

**Figure:**
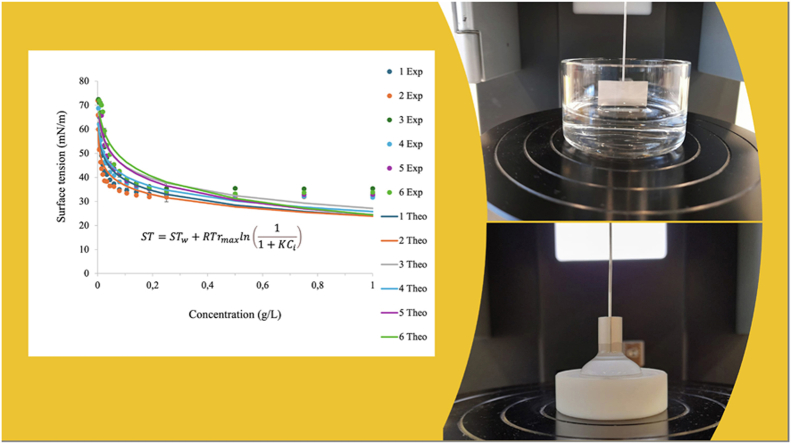

## 1 Introduction

Surfactants are amphiphilic molecules, possessing both hydrophilic and hydrophobic fractions, with properties like those of synthetic surfactants, that enable them to interact at the interfaces of aqueous and non-aqueous solutions [1]. The most relevant property of surfactants is their ability to reduce the surface tension (ST) of water, thereby facilitating the solubilisation and stabilization of active compounds in aqueous solutions. When surfactants are produced by living microorganisms such as yeasts, bacteria, and filamentous fungi, they are referred to as biosurfactants. Microorganisms can produce biosurfactants either bound to their cell membranes, as reported in certain *Bacillus* strains, or excreted into the fermentation media [2,3]. Furthermore, in contrast to synthetic and bio-based surfactants, biosurfactants can be produced through the fermentation of renewable biomass. This presents an advantage in the development of the circular economy, as renewable biomass derived from agri-food streams can serve as raw material. Additionally, biosurfactants are biodegradable, less toxic, and more environmentally friendly [2,4,5]. Hence, there is an increasing interest in the physical properties of biosurfactants [6] and in their antimicrobial activity [7], as well as an emerging interest in obtaining more biodegradable and sustainable formulations. Moreover, Li et al. [8] developed a rhamnolipid-monoethanol amide system for oil recovery. The primary challenge in biosurfactant production, compared to chemical synthesis, lies in the purification process and in the lower productivities and yields as they are secondary metabolites [9,10]. It is therefore essential to employ equipment capable of working with smaller sample quantities to determine whether a fermented extract contains biosurfactants. For instance, commercial surfactin, used as a standard during the production of lipopeptide biosurfactants, has a market price of approximately €23.40 per milligram [11,12].

On the other hand, the surface tension is defined as the force exerted in the plane of the surface per unit length [13]. In a liquid, molecules experience attraction due to cohesive forces. As a result, molecules within the liquid are surrounded by attractive molecules. In contrast, those located at the surface of the liquid, at the interface, are only attracted inward and sideways, leading to a net force directed towards the interior of the liquid. This phenomenon is responsible for surface tension [14,15]. Consequently, the formation of new surfaces is energetically unfavourable, which drives the tendency to minimize their creation, thereby reducing the exposed surface. This behaviour is notably observed in the spherical shape of liquid droplets [16]. Surface tension increases as intermolecular attraction intensifies, and molecular size decreases. While the surface tension of water is approximately 72 mN/m, for other substances like mercury, it can be 500 mN/m, and 34.3 mN/m for tripalmitin [[17], [18], [19]].

One of the methods employed to measure surface tension is the Wilhelmy plate method, first introduced by the German chemist Ludwig Wilhelmy in 1863 [20]. This method involves using a rectangular-shaped plate with known dimensions suspended on a precision balance. An advantageous feature of this method in contrast to others is its exemption from corrections for Archimedes' buoyancy. In the current measurement set-up, the plate remains unsubmerged in the liquid, eliminating the need for such adjustments [21].

The aim of this work is to evaluate the surface tension capacity of three commercial glycolipid biosurfactants in terms of water ST reduction and CMC using the Wilhelmy method that involves a platinum plate with i) different geometry (rectangular or cylindrical) with the same area of contact (4.4 cm^2^), ii) different volumes of biosurfactant solution (2, 4, and 20 mL), and iii) different containers, one made of glass and another made of polytetrafluoroethylene (PTFE), in order to detect similarities or differences between the evaluated conditions in terms of surface tension measurements. Then, the variation of ST with biosurfactant concentration was studied based on the isotherm model proposed by Li & Lu [22] and based on the model proposed by Szyszkowski [23]. Moreover, a correlation statistical analysis should be applied in the study involving Pearson [24] and Spearman [25] coefficients that allows the detection of similarities and differences between pairs of conditions being compared in terms of surface tension.

## 2 Experimental

### 2.1 Commercial biosurfactants

The commercial biosurfactants used for the analysis were Rhamnolipid 90 % pure (from AGAE Technologies LLC company, Corvallis, Oregon, USA), Lactonic (di-acetylated) Sophorolipid (from Biosynth company, Staad, Switzerland), and Rhamnolipid 30 % (from MarkNature company, Pinehurst Ct Fullerton, CA, USA).

According to their technical data sheet, the biosurfactant contained in rhamnolipid 30 % was produced by *Pseudomona aeruginosa* and is composed of rhamnose and C-10 fatty acids, it is soluble in water and possesses a molecular weight of 650.79 Da. Similarly, rhamnolipid 90 % is also composed of a biosurfactant based on rhamnose and C-10 fatty acids with a molecular weight of 650.79 Da. In addition, the evaluated sophorolipid is composed of lactic acid-derived compounds, containing sophorose and C-18 fatty acids with a molecular weight of 688.8 Da.

These biosurfactant extracts were dissolved in Milli-Q water, at room temperature, with an initial concentration of 1 g/L, and then were further serially diluted with Milli-Q water to obtain their respective critical micellar concentration (CMC). Below the CMC, the surface tension of solutions containing biosurfactants experiences notable variations with surfactant concentration. Conversely, above the CMC, the surface tension either remains relatively constant or exhibits changes with a reduced slope, characterized in this stage by the formation of micelles [26].

### 2.2 Elemental analysis of the biosurfactants

Elemental analysis of carbon, hydrogen, and nitrogen was carried out by means of an elemental analyzer (Fisons Carlo Erba EA-1108 CHNS-0, LabX, Midland, ON, Canada), following the procedure used in previous works [27].

### 2.3 Evaluation of ST and CMC of biosurfactants extracts using Wilhelmy plate method

Solutions of commercial biosurfactants consisting of the glycolipids described above were subjected to different dilutions, and ST measurements were obtained at different concentrations. Subsequently, the CMC of each biosurfactant was calculated. The measurements of ST were carried out with a K20 Easy Dyne Tensiometer supplied by Kruss GmbH (Hamburg, Germany), allowing the use of the Wilhelmy plate method. This tensiometer is equipped with two sets of platinum plates: PL21 (rectangular) and PL22 (cylindrical), hereafter referred to as P1 and P2, respectively; and two containers for large (glass) and small (PTFE) volumes. Different assays were thus carried out, in triplicate, with the biosurfactants extracts (rhamnolipid 30 %, rhamnolipid 90 %, and sophorolipid) using different platinum plates in terms of geometry (rectangular or cylindrical), different sample volumes (2, 4, and 20 mL) and different types of container, either glass with a maximum volume of 45 cm^3^ or polytetrafluoroethylene (PTFE) with a maximum volume of 5 cm^3^ as independent variables following the description included in Table 1. Each surface tension data point was recorded until three consecutive measurements showed consistent results. Multiple measurements were taken from the same sample until stability was achieved. Measurements were conducted every 90 s.

**Table 1:** Experimental design used in this study varying the volume, plate model, and container.

**Assay**	**Volume (mL)**	**Plate**	**Container**
**1**	4	P1	Glass
**2**	20	P1	Glass
**3**	4	P2	PTFE
**4**	4	P2	Glass
**5**	4	P1	PTFE
**6**	2	P2	PTFE

Note: PL21 referred as P1 is rectangular; PL22 referred as P2 is cylindrical.

Once the ST curve was established, the CMC was graphically determined where the baseline of minimal surface tension aligns with the slope indicating a linear decline in surface tension. Moreover, below the CMC, the data were adjusted to a linear equation (Equation (1)) that can predict the concentration of biosurfactant for a specific *ST* value. Where *ST* is the experimental ST value (mN/m); C_*eq*_ is the concentration of biosurfactant in equivalents of the biosurfactant used to obtain this equation (g/L); m is the slope of the data after applying least Squares ((mN/m)/(g/L)); and A holds a constant value of 72 mN/m, representing the ST_w_ of water when the biosurfactant concentration is zero.

Equation 1 $$C_{eq}=\frac{{ST}_{w}-A}{m}$$

in addition, the variation of *ST* values with the concentration of biosurfactant was studied using Equation (2) based on the model proposed by Li & Lu [22]**,** and based on the model proposed by Szyszkowski [23] where a_i_, defined as the activity of component i, was replaced by C_i_, the concentration of biosurfactant (g/L).

Equation 2 $$ST={ST}_{w}+RTr_{\max}\;\ln\left(\frac{1}{1+KC_{i}}\right)$$

where ST is the surface tension of the sample (mN/m); ST_w_ is the surface tension of water with a constant value of 72 mN/m; R is the gas constant with a value of 8.31 at J/mol K; T is the temperature in K; r_max_ is the maximum number of biding sites (mol/m^2^); C_i_ is the concentration of biosurfactant (g/L); and K is the adsorption equilibrium constant that defines the variation of ST with concentration of biosurfactant.

Moreover, to check the accuracy of the model, a non-commercial biosurfactant extract obtained from a residual stream, generated during fermented butter milk production provided from the dairy company Feiraco (Spain), was evaluated under the conditions of Assay 1 (Table 1). To obtain the biosurfactant extract, the residual stream was filtered with a 45 μm hydrophilic PTFE syringe filter (Filter-Lab), after which it was freeze-dried using a lyophilizer LyoQuest (Telstar). After that, the biosurfactant extract was diluted at 1 g/L and serial dilutions were made and surface tension was measure at different concentrations following the procedure described in section 2.3.

**Fig. 1:**
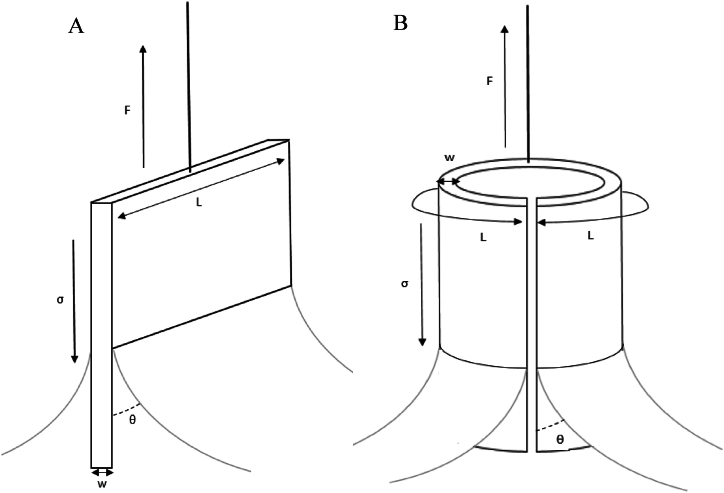
Wilhelmy plate method and force balance with A) rectangular plate or B) cylindrical plate.

To perform the analysis, the plate is partially immersed in the liquid of unknown surface tension, wetting it so that when a vertical force is applied to remove it from the liquid, a curved interface forms on its surface [27]. Now, when the plate is in the position just before it starts to be extracted from the liquid, the balance of forces acting on the plate can be calculated. These forces include the surface tension, which attracts the plate towards the liquid, and the force applied to raise the plate, which keeps the plate stationary at the time of measurement (Fig. 1) [28,29]. Fig. 1 represents the forces per unit length on the different platinum plates used: rectangular (Fig. 1A) or cylindrical (Fig. 1B).

Considering that the thickness of the plate (w = 0.20 mm) is smaller than its length (L = 19.90 mm) and taking into account that the plate is typically made of smooth and high free-energy materials like platinum, which allows the contact angle (θ) to be considered as zero, it can be concluded that the surface tension will be directly proportional to external forces, and inversely proportional to the length of the plate [21].

### 2.4 Statistical treatment of data

ST data used to calculate the CMC for each biosurfactant were subjected to a correlation study by pairs and the correlation factors of Pearson (P_P_) and Spearman (P_S_) were obtained. Each ST pair being compared was carried out at the same concentration of biosurfactant. The correlation factors (P_P_ and P_S_) were calculated using the statistical correlation factors proposed by Henschel et al. [30] following Equation 3, Equation 4 respectively. P_P_ was estimated directly with the Excel statistical tool as this calculation is incorporated in the data analysis package (Equation (3)), whereas P_S_ was obtained using Equation (4).

Equation 3 $$P_{P}=\frac{\sum_{i}\left(xi-\bar{x}\right)-\left(\text{yi}-\bar{y}\right)}{\sqrt{\sum {\left(xi-\bar{x}\right)}^{2}}\sqrt{{\left(yi-\bar{y}\right)}^{2}}}$$

In Equation (3), x_i_ and y_i_ are the elements of the intensity vectors representing ST value under comparison (mN/m) and $\bar{x}$ and $\bar{y}$ the mean values of the x_i_ and y_i_ values (mN/m), respectively.

Equation 4 $$P_{S}=1-\frac{6\;\sum_{i}d_{i}^{2}}{n\left(n^{2}-1\right)}$$

in Equation (4), d_i_ is the difference between the ranks of the ST values compared in their respective data set and n the number of elements in each vector to be compared.

## 3 Results and discussion

### 3.1 Elemental analysis of the biosurfactants under evaluation

The surface tension capacity of a biosurfactant extract is closely related to its composition. Thus, Table S1 (see supplementary material) includes the elemental composition of the three biosurfactants under study, observing that rhamnolipid 30 % and 90 % differ in the percentage of C, rhamnolipid 90 % being closer to sophorolipid than rhamnolipid 30 %. In addition, it was observed that both rhamnolipids contain a higher percentage of N (around 1.1 %) than sophorolipid (<0.08 %), probably due to the presence of peptides or proteins in their molecular composition, whereas the percentage of H in the sophorolipid is higher (8.20 %)

### 3.2 Effect of tensiometer plate geometry, type of container, and volume of sample on the surface activity analysis of biosurfactants

**Fig. 2:**
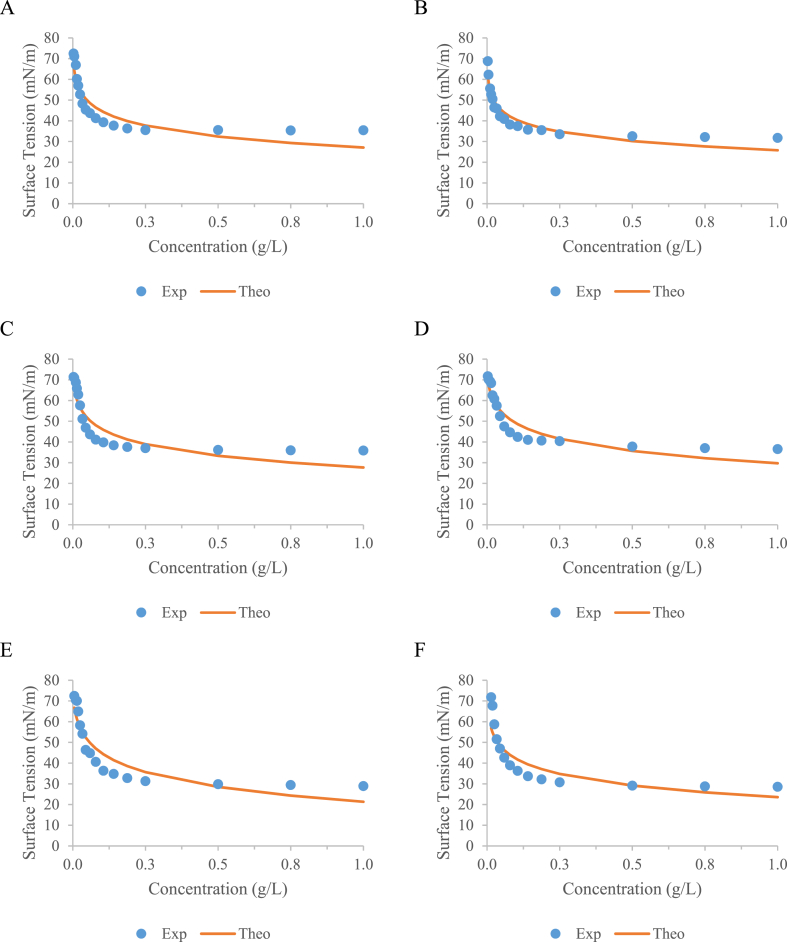
Surface tension values obtained with decreasing concentrations of biosurfactant extract for A) rhamnolipid 90 % Assay 3, B) rhamnolipid 90 % Assay 4, C) sophorolipid, Assay 3 D) sophorolipid, Assay 4, E) rhamnolipid 30 % Assay 3, and F) rhamnolipid 30 % Assay 4.

To evaluate whether the geometry of platinum plate, volume of sample, and container material affect the ST value and CMC of water solution containing different types of biosurfactants (rhamnolipid 30 %, rhamnolipid 90 %, and sophorolipid). Fig. 2 includes the variation of ST regarding the concentration of these surface-active agents for different assays shown above in Table 2 fitted to Equation (2) based on the model proposed by Li & Lu [22] and based on the model proposed by Szyszkowski [23].

**Table 2:** Fit parameters obtained by applying the modified Li & Lu model [22].

**Biosurfactant**	**Assay**	**r**_**max**_**(mol/m**^**2**^**)**	**K**	**r**^**2**^
**Rhamnolipid 90 %**	1	2.689 × 10^−6^	1548.221	0.790
	2	2.273 × 10^−6^	5951.224	0.836
	3	3.163 × 10^−6^	339.768	0.855
	4	2.649 × 10^−6^	1295.936	0.908
	5	3.615 × 10^−6^	220.423	0.837
	6	4.134 × 10^−6^	112.343	0.837
**Sophorolipid**	1	2.300 × 10^−6^	2166.983	0.781
	2	2.287 × 10^−6^	2910.068	0.764
	3	3.363 × 10^−6^	223.182	0.849
	4	3.548 × 10^−6^	132.595	0.889
	5	3.986 × 10^−6^	106.715	0.780
	6	4.209 × 10^−6^	75.380	0.786
**Rhamnolipid 30 %**	1	2.949 × 10^−6^	731.840	0.882
	2	2.711 × 10^−6^	1432.999	0.862
	3	4.315 × 10^−6^	123.246	0.866
	4	3.341 × 10^−6^	384.336	0.820
	5	3.956 × 10^−6^	174.986	0.830
	6	3.780 × 10^−6^	147.539	0.866

Regarding the geometry of the platinum plate, it is important to point out that despite the change in the shape of the P1 and P2 plates, their length and thickness dimensions remain constant. However, this change in geometry could potentially impact the obtained values. This could occur either by altering the arrangement of the plate's side surfaces, or because the inner circle surface of the plate might provide an area in which the liquid being analysed can become trapped due to the adhesive and cohesive forces of water. These forces are the same ones that allow water to rise in the xylem of plants or in capillary tubes [31]. Considering that the contact angle with the platinum plate is 0°, the higher the dilution of the biosurfactant extract sample, the higher its ST value should be. Consequently, one might expect a greater height to which the solution could ascend, potentially affecting its wettability and, thus, the results obtained. It should also be noted that there is a minimum distance of approximately 5 mm between the plate and the container [32] to prevent the formation of a capillary bridge, which could impact the results by causing the wall effect [33]. Therefore, in assay 5, the geometry of plate P1 could affect the results as it is closer to the edges of container P2 compared to cases where the corresponding plates and containers are used together. However, the results obtained differ to a certain extent from this possibility. Regardless of the commercial biosurfactant used, their ST values remain similar in the six conditions summarized in Table 1.

Moreover, CMC values were obtained for the three biosurfactants under evaluation and for all the conditions tested (see Table 3). CMC can be defined as the concentration at the point of intersection in the graph where the ST stops decreasing with increasing concentration of surfactant, after that ST it is almost the same. The choice of the exact point of intersection has a certain subjectivity and very small changes in the wide region between them can give variation in CMC [34].

**Table 3:** CMC values obtained for the evaluated biosurfactants under different conditions of surface tension measurement.

**Biosurfactant**	**Assay**	**Min ST (mN/m)**	**Mean ST (mN/m)**	**CMC (g/L)**	**Mean CMC (g/L)**
Rhamnolipid 90 %	1	32.50	32.75 ± 0.35	0.047	0.050 ± 0.007
	2	33.00		0.043	
	3	35.40		0.047	
	4	31.70		0.049	
	5	32.95		0.050	
	6	33.85		0.064	
Sophorolipid	1	35.55	35.75 ± 0.55	0.056	0.064 ± 0.008
	2	35.00		0.056	
	3	35.80		0.061	
	4	36.55		0.068	
	5	35.45		0.073	
	6	36.15		0.072	
Rhamnolipid 30 %	1	28.10	28.67 ± 0.65	0.080	0.089 ± 0.012
	2	27.85		0.076	
	3	28.90		0.084	
	4	28.55		0.094	
	5	28.95		0.100	
	6	29.65		0.104	

In this case, the distribution of ST values versus concentration is very homogenous for all the sceneries tested (see Fig. 2), with CMC values ranging from 0.043 to 0.064 g/L for rhamnolipid 90 %, 0.056–0.073 g/L for sophorolipids, and 0.076–0.104 g/L for rhamnolipid 30 %. A higher CMC was observed for rhamnolipid 30 %, which is coherent with the grade of purity, whereas sophorolipid gave intermediate CMC values. If minimum ST values are compared, rhamnolipid 30 % gave the lowest value, which varied between 27.85 and 29.65 mN/m (mean value 28.67 ± 0.65 mN/m), whereas the highest surface tension reduction values were achieved with sophorolipids, which varied between 35.00 and 36.55 mN/m (mean value 35.75 ± 0.55 mN/m). Meanwhile, rhamnolipid 90 % gave intermediate surface tension values between 31.7 and 35.4 mN/m (mean value 32.75 ± 0.35 mN/m). Rhamnolipid 30 % probably contains some impurity from the biosynthesis or from the downstream process that produced a synergy effect promoting a higher surface tension reduction in comparison with rhamnolipid 90 %, although this reduction in surface tension is less consistent than that produced by rhamnolipid 90 % as rhamnolipid 30 % gave higher CMC under all the conditions tested.

Furthermore, regarding the minimum surface tension value obtained for each of the biosurfactants under different conditions, it was confirmed that these aligned with the expected results regarding the surface tension activity of the biosurfactants under evaluation [[35], [36], [37]]. Thus, Pal et al. [35] have reported that sophorolipids at room temperature can reduce the surface tension of water to 35 mN/m; whereas Thakur et al. [36] have published that rhamnolipids reach a surface tension of approximately 30 mN/m.

**Fig. 3:**
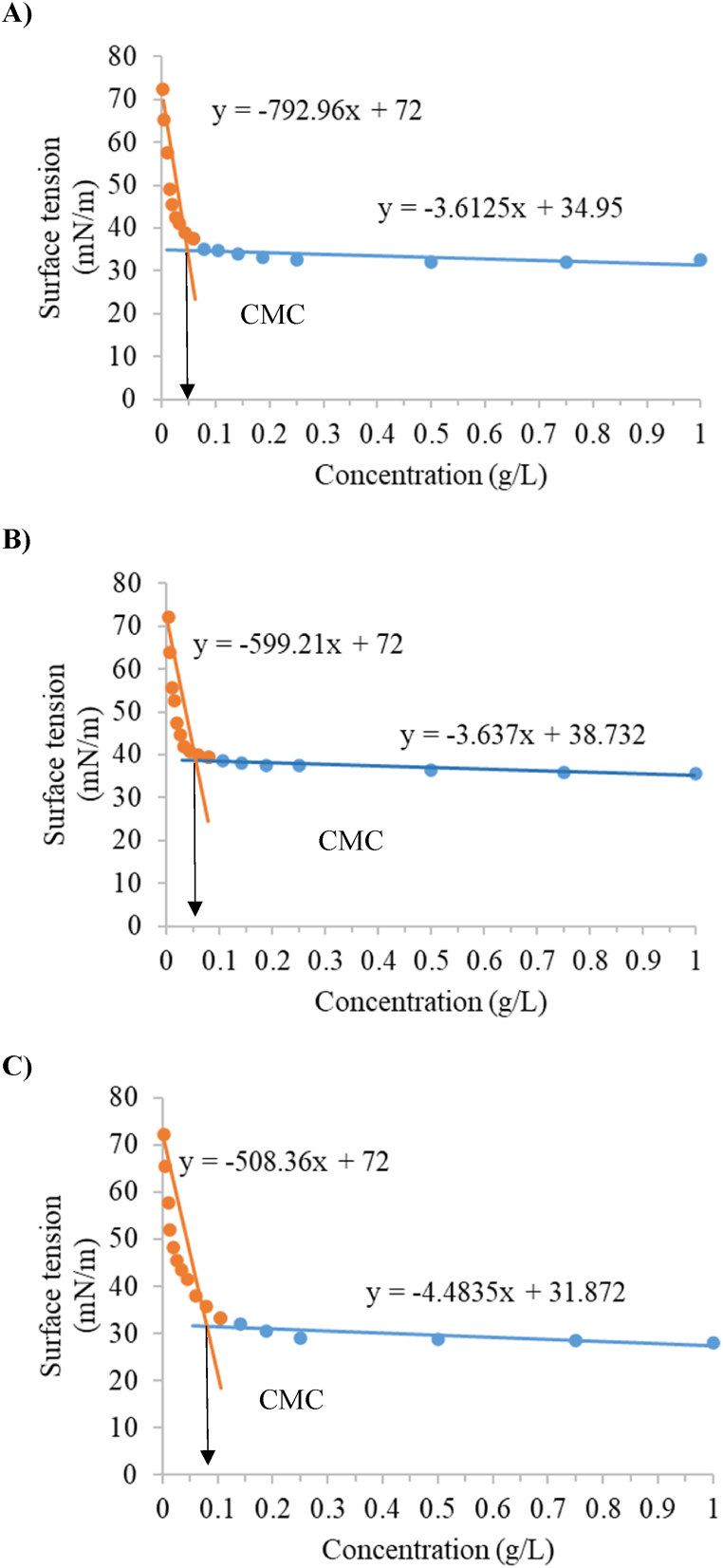
CMC values for: A) rhamnolipid 90 %; B) sophorolipid; and C) rhamnolipid 30 % using the conditions of assay 1.

As an example, Fig. 3 includes the adjustment of surface tension to concentration of the three biosurfactants under evaluation by two straight lines for assay 1, using the rectangular plate with a volume of biosurfactant solution of 4 mL and using the glass container with a capacity of 45 cm^3^ (see Table 1).

Additionally, Table 2 includes the fit parameters r_max_ and K of the model proposed, obtaining coefficients of determination (r^2^) between 0.764 and 0.908 for the different sceneries proposed.

### 3.3 Surface tension stability

**Fig. 4:**
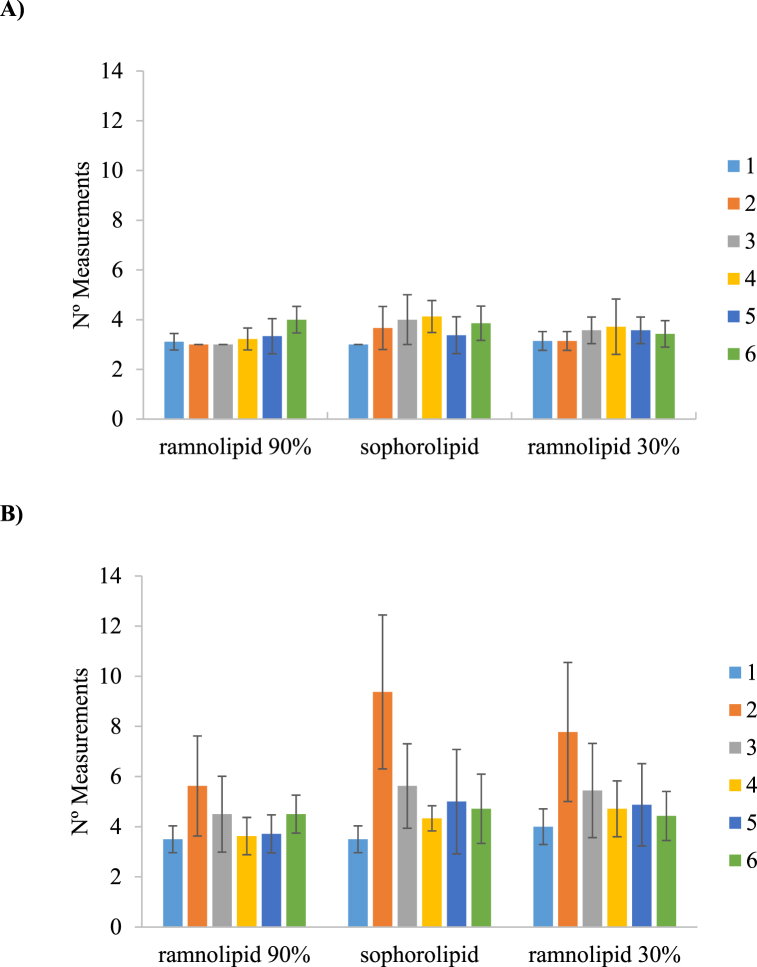
Number of measurements required to achieve ST values with decreasing concentrations of biosurfactant extract: A) over CMC and B) under CMC.

Based on the number of measurements needed to achieve result stability, when surface tension is measured with concentration values of biosurfactant extract of 1 g/L up to CMC (0.043–0.104 g/L) the different conditions remain relatively similar, regardless of the commercial brand involved (Fig. 4A). However, when concentration values fall under CMC, an increase in the number of measurements required is observed, especially with assay 2 (Fig. 4B). It can be speculated that near the CMC there is a mix of biosurfactant molecules forming micelles and others not forming micelles, leading to greater surface tension instability which can be more noticeable in higher volumes as this behaviour was detected systematically in the higher volume of 20 mL.

Other comparable measurement equipment utilizing the Wilhelmy Plate method has demonstrated the requirement for multiple measurements or extended waiting times to attain result stability when dealing with solutions containing surfactants [38,39], which would explain the need for further measurements at some concentrations, while at others the final value is obtained from the beginning.

### 3.4 Comparative statistical study

To determine whether the obtained surface tension values with different commercial biosurfactants are indeed similar, the value profiles obtained were compared using the Pearson correlation coefficient (Table 4), where it was observed that the lowest of the values is 0.91, so they can indeed be considered very similar. To double-check this comparison, the Spearman correlation coefficient was also employed (Table 4), where the lowest of the obtained results was 0.93, thus confirming the similarity of the results obtained under different conditions for all three commercial biosurfactants. These results are consistent with the behaviour of data when an isotherm model was proposed.

**Table 4:** Pearson (P_p_) and Spearman (P_S_) correlation coefficient values obtained with different commercial samples under the chosen experimental conditions.

Rhamnolipid 90 %						
**Assay**	1 (P_p_/P_S_)	2 (P_p_/P_S_)	3 (P_p_/P_S_)	4 (P_p_/P_S_)	5 (P_p_/P_S_)	6 (P_p_/P_S_)
1	1.00/1.00	0.99/0.96	0.98/0.98	0.98/0.98	0.96/1.00	0.92/1.00
2	0.99/0.96	1.00/1.00	0.99/0.94	0.99/0.93	0.98/0.95	0.95/0.95
3	0.98/0.98	0.99/0.94	1.00/1.00	0.97/0.99	0.99/0.99	0.98/0.98
4	0.98/0.98	0.99/0.93	0.97/0.99	1.00/1.00	1.00/0.99	0.98/0.99
5	0.96/1.00	0.98/0.95	0.99/0.99	1.00/0.99	1.00/1.00	0.99/1.00
6	0.92/1.00	0.95/0.95	0.98/0.98	0.98/0.99	0.99/1.00	1.00/1.00
**Sophorolipid**						
**Assay**	1 (P_p_/P_S_)	2 (P_p_/P_S_)	3 (P_p_/P_S_)	4 (P_p_/P_S_)	5 (P_p_/P_S_)	6 (P_p_/P_S_)
1	1.00/1.00	0.99/1.00	0.97/1.00	0.96/1.00	0.96/1.00	0.91/1.00
2	0.99/1.00	1.00/1.00	0.99/0.99	0.97/0.99	0.98/0.99	0.94/0.99
3	0.97/1.00	0.99/0.99	1.00/1.00	0.99/1.00	0.99/1.00	0.97/1.00
4	0.96/1.00	0.97/0.99	0.99/1.00	1.00/1.00	1.00/1.00	0.99/1.00
5	0.96/1.00	0.98/0.99	0.99/1.00	1.00/1.00	1.00/1.00	0.98/1.00
6	0.91/1.00	0.94/0.99	0.97/1.00	0.99/1.00	0.98/1.00	1.00/1.00
**Rhamnolipid 30 %**						
**Assay**	1 (P_p_/P_S_)	2 (P_p_/P_S_)	3 (P_p_/P_S_)	4 (P_p_/P_S_)	5 (P_p_/P_S_)	6 (P_p_/P_S_)
1	1.00/1.00	0.99/1.00	0.99/1.00	1.00/1.00	0.98/0.98	0.98/0.99
2	0.99/1.00	1.00/1.00	1.00/1.00	1.00/1.00	0.98/0.98	0.97/0.99
3	0.99/1.00	1.00/1.00	1.00/1.00	0.99/1.00	0.99/0.98	0.97/0.99
4	0.99/1.00	1.00/1.00	1.00/1.00	1.00/1.00	0.99/0.98	0.96/0.99
5	0.98/0.98	0.98/0.98	0.99/0.98	0.99/0.98	1.00/1.00	0.98/0.99
6	0.98/0.99	0.97/0.99	0.97/0.99	0.96/0.99	0.98/0.99	1.00/1.00

### 3.5 Application of an isotherm model to a non-commercial biosurfactant extract

**Fig. 5:**
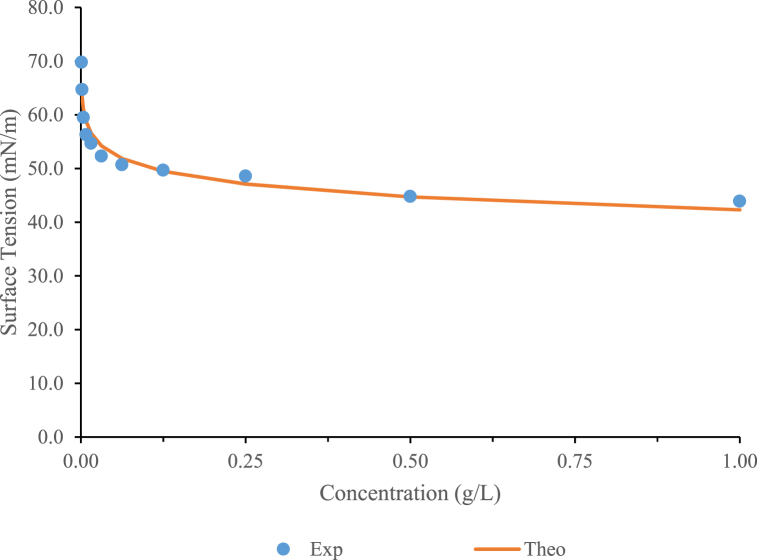
Variation of surface tension with the concentration that fits the isotherm model proposed by Li & Lu [22].

When butter is produced through a fermentation stage, lactic bacteria convert sugars into lactic acid and other prebiotic substances such as lactic acid bacteria are considered probiotic microorganisms with the capacity to produce biosurfactants. Thus, the buttermilk stream, obtained during the elaboration of culture butter involving lactic acid bacteria, can be a potential source of glycolipopeptide or glycopeptide biosurfactants [40,41]. Fig. 5 shows the isotherm variation of concentration of biosurfactant extract obtained from butter residual stream with the surface tension using the protocol established in assay 1, volume 4 mL, and rectangular plate. This extract was able to reduce the surface tension of water by more than 20 units, with a CMC of 0.031 g/L, being more favourable than other CMCs reported in the literature [42]. The coefficient of determination (r^2^) obtained with the isotherm model proposed by Li and Lu [22] was 0.93, and the fit parameters r_max_ and K were 1.42 × 10^−6^ (mol/m^2^) and 2191.26, respectively.

## 4 Conclusions

Overall, this work offers the first insight into biosurfactant surface activity assessments by evaluating the influence of tensiometer plate geometry and sample volume. The results obtained suggest that there are no substantial differences in ST and CMC values when measuring biosurfactant solutions, regardless of variations in plate geometries, container materials, and sample volumes. Using 20 mL of sample not only entails higher costs due to the larger sample volume but also requires more investment. Using 4 mL, whether with the high-volume or low-volume kit, yields similar results with virtually the same number of measurements between them, making it possible to use either depending on biosurfactant availability. However, this similarity is only achievable because the volume used, 4 mL, falls within the working range of both kits. The minimum working volume of the high-volume kit is approximately 3 mL, as using a smaller volume causes the plate to collide with the container, preventing proper submersion. The maximum working volume of the low-volume kit is 5 mL, so when volumes fall outside these ranges, working with the corresponding kit becomes essential. Moreover, reducing the sample volume to approach the stability limit of the liquid surface shape may affect the accuracy of the results more than the choice of kit. Finally, the isotherms that show the variation of concentration with surface tension fit the model proposed by Li and Lu, this being the first time that these isotherm models have been applied to biosurfactants. Additionally, to enhance the accuracy of the proposed model, a non-commercial biosurfactant extract, produced from a dairy waste stream, was included in the study, further emphasizing the innovative nature of this work. Nevertheless, additional experiments are required to confirm this fitting for other biosurfactant extracts.

## CRediT authorship contribution statement

**N. Russo-Martínez:** Writing – original draft, Visualization, Methodology, Investigation, Formal analysis, Conceptualization. **X. Vecino:** Writing – review & editing, Visualization, Supervision, Project administration, Investigation, Funding acquisition, Formal analysis, Conceptualization. **A.B. Moldes:** Writing – review & editing, Writing – original draft, Visualization, Validation, Resources, Investigation, Formal analysis, Conceptualization. **J.M. Cruz:** Writing – review & editing, Visualization, Supervision, Resources, Project administration, Investigation, Funding acquisition, Formal analysis, Conceptualization.

## Declaration of competing interest

The authors declare that they have no known competing financial interests or personal relationships that could have appeared to influence the work reported in this paper.
