# Supplementary material for: Modelling and impact of tensiometer plate geometry and sample volume on biosurfactant surface activity assessment
Source: Heliyon. 2024 Sep 23;10(19):e38325. doi: 10.1016/j.heliyon.2024.e38325 (PMC11470507; doi:10.1016/j.heliyon.2024.e38325)
Supplement: Multimedia component 1 [file mmc1.docx]

**Modelling and Impact of Tensiometer Plate Geometry and Sample Volume on Biosurfactant Surface Activity Assessment**

N. Russo-Martínez, X. Vecino, A.B. Moldes*, J.M. Cruz

Chemical Engineering Department, School of Industrial Engineering – CINTECX, University of Vigo, Campus As Lagoas-Marcosende, 36310 Vigo, Spain.

*Corresponding author: amoldes@uvigo.gal

**Table S1**. Elemental composition, CHNS, of the three commercial biosurfactants under study.

| **Biosurfactants** | **C (%)** | **H (%)** | **N (%)** | **S (%)** |
| --- | --- | --- | --- | --- |
| Rhamnolipid 90% | 58.48 ± 0.54 | 7.57 ± 0.39 | 1.08 ± 0.11 | < 0.30 |
| Sophorolipid | 59.32 ± 0.08 | 8.20 ± 0.19 | < 0.08 | < 0.30 |
| Rhamnolipid 30% | 27.48 ± 0.04 | 7.04 ± 0.13 | 1.11 ± 0.01 | < 0.30 |
